# Vitamin D Receptor gene polymorphisms and plasma levels are associated with lumbar disc degeneration

**DOI:** 10.1038/s41598-019-44373-2

**Published:** 2019-05-24

**Authors:** Qinghua Yang, Yang Liu, Yewen Guan, Xinli Zhan, Zengming Xiao, Hua Jiang, Qingjun Wei

**Affiliations:** 1 The First Affiliated Hospital of Guangxi Medical University, Division of Spine Surgery, Nanning, 530021 China; 2 The First Affiliated Hospital of Guangxi Medical University, Department of Orthopedic Surgery, Nanning, 530021 China

**Keywords:** Microsatellite instability, Predictive markers

## Abstract

The purpose of this study was to investigate the association of Vitamin D Receptor (VDR) gene polymorphisms and VDR levels with lumbar disc degeneration (LDD). TaqMan SNP Genotyping Assay was utilized to probe VDR gene polymorphisms including the *FokI* (rs2228570), *ApaI* (rs7975232) and *TaqI* (rs731236) in 454 patients with LDD and 485 controls. Enzyme-Linked Immunosorbent Assay (ELISA) was used to detect plasma VDR levels. The patients with LDD were divided into three subgroups (subgroup 1: lumbar disc herniation; subgroup 2: lumbar spinal stenosis; subgroup 3: lumbar spondylolisthesis) to further probe the association of plasma VDR levels and VDR gene polymorphisms and LDD. Moreover, immunohistochemistry (IHC) was implemented to evaluate VDR expression in lumbar degenerated disc and normal disc. Allele and genotype frequency of *TaqI* (rs731236) were significantly different in patients with LDD and controls (all P < 0.05). For *TaqI* polymorphism, the frequencies of T allele were significantly higher in the LDD patients compared with controls (OR = 1.319; 95%CI 1.091 to 1.595; P = 0.004, adjusted (OR = 1.319; 95%CI 1.091 to 1.595; P = 0.004, adjusted OR = 1.383; 95%CI 1.135 to 1.684; P = 0.016). Furthermore, the allele distribution showed a higher frequency of the T allele in the patients with lumbar disc herniation in subgroup 1 (OR = 1.384; 95% CI 1.105 to 1.732; P = 0.004, adjusted OR = 1.319; 95%CI 1.091 to 1.595; P = 0.016). Plasma VDR levels and VDR expression were significantly lower in patients with LDD compared with controls (all P < 0.05). Moreover, the TT genotype of *TaqI* polymorphism was significantly associated with lower plasma VDR levels in patients with LDD (P = 0.002). *TaqI* (rs731236) polymorphism was associated with a predisposition to LDD. Plasma VDR and VDR expression levels may be the marker for the occurrence and development of LDD.

## Introduction

Low back pain is a very common musculoskeletal disorder in most developed and developing countries, which could result in an increasing global burden of disease^1,2^. Lumbar disc degeneration (LDD) may be the top cause of low back pain^3,4^. LDD presents with a complex cascade of lumbar spine and its accessories degenerative changes. Although the exact etiology is not fully understood, it is widely accepted that LDD result from the combined effects of multiple genes and environmental factors^5,6^. Growing evidence suggested that LDD may be explained primarily by genetic factors^5,7^. Several genetic risk factors have been identified, including genes coding for Vitamin D Receptor (VDR), Collagen IX, and Collagen XI^8^. VDR gene is currently one of the most studied candidate genes associated with a predisposition to LDD. VDR is a member of the steroid and thyroid hormone receptor family of ligand-activated transcription factors that mediates the genomic effects of 1, 25-dihydroxyvitamin D in a wide variety of tissues^9,10^. The VDR has been proved to play a crucial role in regulating chondrocytes proliferation and differentiation, matrix production, and apoptosis^11–13^.

Several variants of the VDR gene including the *FokI* (rs2228570), *ApaI* (rs7975232) and *TaqI* (rs731236) have been found to be associated with LDD^14–23^. The *ForkI* polymorphism is located in the start codon of VDR gene. Several studies showed that the short 424 amino acid VDR protein variant encoded by the “F” allele is more active than the long 427 amino acid variant encoded by the “f” allele^24–26^. The *ApaI*, and *TaqI* polymorphisms are located near the 3′ untranslated region (UTR) of the VDR gene^27,28^. It is known that 3′ UTR involves the process of gene expression by affecting gene localization, stability and translation of mRNAs^29,30^. However, the influence of VDR gene polymorphisms on VDR protein function remain not fully understood. Moreover, previous studies have produced conflict results with regards to the association between VDR gene polymorphisms and LDD^14–24^. It is important to further study the association between VDR gene polymorphisms and LDD in different ethnic populations^15,17,31^. The purpose of this study was to investigate the association of VDR gene polymorphisms including the *FokI* (rs2228570), *ApaI* (rs7975232) and *TaqI* (rs731236) susceptibility to LDD in Chinese southern population. Furthermore, we used Enzyme-Linked Immunosorbent Assay (ELISA) and immunohistochemistry (IHC) to evaluate VDR expression levels in plasma and lumbar disc tissue between LDD patients and controls.

## Results

### Demographic data

The characteristics of the overall population of cases and controls are summarized in Table 1. There was no significant difference in age, gender, BMI, smoking habit between the case and control groups. The median age of control group was 54.2 years, ranging from 15.6 to 62.4 years. The median age of case group was 51.6 years, ranging from 16.8 to 84.4 years. A lower age was found in subgroup 1 compared with control group and the other two subgroups (all P < 0.05). There was no statistical difference between subgroup 2 and 3 (P > 0.05).

**Table 1:** Characteristics of the study subjects between control and case groups including three subgroups.

Characteristics		Control group	Case group^#^	Subgroup 1	Subgroup 2	Subgroup 3
Age (years)	(median, years)	54.2 (15.6–62.4)	51.6 (16.8–84.4)	42.2 (16.8–76.4)*	61.8 (24.3–84.4)	57.3 (24.3–77.5)
Gender	Males, n (%)	252 (52.1)	259 (57.0)	184 (71.0)	54 (20.8)	21 (8.1)
	Females, n (%)	233 (47.9)	195 (43.0)	81 (41.5)	52 (26.7)	62 (31.8)
BMI in kg/m^2^	Mean ± SD	24.15 ± 2.93	24.01 ± 6.37	23.44 ± 3.45	24.10 ± 3.23	24.31 ± 3.53
Past or current smokers	n (%)	175 (36.1)	169 (37.2)	120 (45.3)	35 (33.0)	14 (16.9)

^#^Case group composed of the Subgroup 1, Subgroup 2, and Subgroup 3. *Compared with control group, P value is less than 0.05.

Age is the actual age when participants were included in this study.

Of the subgroup 1, the disc samples from 34 patients were randomly assigned to perform the IHC analysis. The case group of IHC from subgroup 1 included 18 males and 16 females, and the median age was 53.1 years (ranging from 23.8 to 69.2 years). The control group of IHC included 13 males and 8 females, and the median age was 56.2 years (ranging from 24.8 to 76.3 years). The mean BMI was 23.79 ± 3.98 in case group and 21.43 ± 5.34 in control group. No significant difference in age and BMI were observed between the case and control groups.

### VDR gene polymorphisms and LDD susceptibility

The distributions of alleles and genotypes of VDR gene polymorphisms are presented in Table 2. All the three studied SNPs were in the Hardy-Weinberg Equilibrium (HWE) in control group (P > 0.05). The genotype and allele frequencies of *TaqI* polymorphism were significantly different between the patients with LDD and controls (all P < 0.05). After Logistic regression analysis and Bonferroni correction, the frequencies of the T allele of *TaqI* polymorphism were significantly higher in the LDD patients compared with controls (OR = 1.319; 95%CI 1.091 to 1.595; P = 0.004, adjusted OR = 1.383; 95%CI 1.135 to 1.684; P = 0.016). Moreover, there was a significant association between the *TaqI* polymorphism and lumbar disc herniation (subgroup 1) (P < 0.05). The allele distribution showed a higher frequency of the T allele in the patients with lumbar disc herniation (OR = 1.384; 95% CI 1.105 to 1.732; P = 0.004, adjusted OR = 1.420; 95%CI 1.151 to 1.842; P = 0.016). No significant association of *TaqI* polymorphism with lumbar spinal stenosis (subgroup 2) and lumbar spondylolisthesis (subgroup 3) were observed (all P > 0.05). Neither the genotype nor the allele frequencies of *FokI* and *ApaI* polymorphisms were significantly different between case group including the subgroups and the control group (all P > 0.05). Furthermore, the result remained stable after adjustment for age (Table 2).

**Table 2:** Allele and genotype distributions of *ForkI* (rs2228570), *ApaI* (rs7975232) and *TaqI* (rs731236) in control and case groups or subgroups.

Polymorphisms	Genotype (%)					Allele (%)						
*FokI* (rs2228570)	AA (ff)	AG (Ff)	GG (FF)	P(χ2)	P*	A (f)	G (F)	P(χ2)	P*	OR (95% CI)	Adjusted OR^#^(95% CI)	HWE
Controls (n = 485)	126 (26.0)	225 (46.4)	134 (27.6)			477 (49.2)	493 (50.8)					0.113
Cases (n = 454)	122 (26.9)	207 (45.6)	125 (27.5)	0.949	0.999	451 (49.7)	457 (50.3)	0.830	0.999	0.980 (0.818–1.175)	1.021 (0.851–1.222)	0.061
Subgroup1 (n = 266)	76 (28.6)	123 (46.2)	67 (25.2)	0.666	0.999	275 (51.7)	257 (48.3)	0.351	0.999	0.904 (0.732–1.117)	1.108 (0.894–1.374)	0.227
Subgroup2 (n = 105)	26 (24.8)	48 (45.7)	31 (28.5)	0.918	0.999	100 (47.6)	110 (52.4)	0.683	0.999	1.064 (0.790–1.435)	0.914 (0.687–1.277)	0.683
Subgroup3 (n = 83)	20 (24.1)	36 (43.4)	27 (32.5)	0.657	0.999	76 (45.8)	90 (54.2)	0.419	0.999	1.146 (0.824–1.594)	0.990 (0.626–1.222)	0.250
*ApaI* (rs7975232)	AA (aa)	AC (Aa)	CC (AA)	P (**χ**2)	P*	A (a)	C (A)	P (**χ**2)	P*	OR (95% CI)	Adjusted OR^#^ (95% CI)	HWE
Controls (n = 485)	50 (10.3)	191 (39.4)	244 (50.3)			291 (30.0)	679 (70.0)					0.170
Cases (n = 454)	34 (7.5)	203 (44.7)	217 (47.8)	0.137	0.548	273 (30.1)	635 (69.9)	0.942	0.999	1.007 (0.827–1.228)	1.001 (0.814–1.209)	0.149
Subgroup1 (n = 266)	20 (7.5)	116 (43.6)	130 (48.9)	0.323	0.999	156(29.3)	376 (70.7)	0.784	0.999	1.033 (0.819–1.301)	1.024 (0.750–1.201)	0.395
Subgroup2 (n = 105)	7 (6.7)	50 (47.6)	48 (45.7)	0.225	0.900	64 (30.5)	146 (69.5)	0.891	0.999	0.978 (0.707–1.352)	0.940 (0.745–1.462)	0.205
Subgroup3 (n = 83)	7 (8.4)	37 (44.6)	39 (47.0)	0.643	0.999	51(30.7)	115 (69.3)	0.851	0.999	0.966 (0.676–1.381)	0.978 (0.744–1.534)	0.667
*TaqI* (rs731236)	AA (tt)	AG (Tt)	GG (TT)	P (**χ**2)	P*	A (t)	G (T)	P (**χ**2)	P*	OR (95% CI)	Adjusted OR^#^ (95% CI)	HWE
Controls (n = 485)	63 (13.0)	246 (50.7)	176(36.3)			372 (38.1)	598 (61.9)					0.109
Cases (n = 454)	32 (7.0)	227(50.0)	195 (43.0)	0.004	0.016	291 (32.0)	617 (68.0)	0.004	0.016	1.319 (1.091–1.595)	1.383 (1.135–1.684)	0.001
Subgroup1 (n = 266)	17 (6.4)	131 (49.2)	118 (44.4)	0.007	0.028	165 (31.0)	367 (69.0)	0.004	0.016	1.384 (1.105–1.732)	1.420 (1.151–1.842)	0.013
Subgroup2 (n = 105)	8 (7.6)	54 (51.4)	43 (41.0)	0.275	0.999	70 (33.3)	140 (66.7)	0.173	0.692	1.244 (0.908–1.704)	1.202 (0.878–1.611)	0.107
Subgroup3 (n = 83)	7 (8.4)	42 (50.6)	34 (41.0)	0.446	0.999	56 (33.7)	110 (66.3)	0.257	0.999	1.222 (0.864–1.729)	1.215 (0.872–1.756)	0.230

OR, odds ratio; CI, confidence interval; HWE, Hardy-Weinberg equilibrium; P*, Bonferroni-corrected P value; Adjusted OR^#^(95% CI), OR adjusted for age.

### Plasma VDR levels and LDD

**Figure 1:**
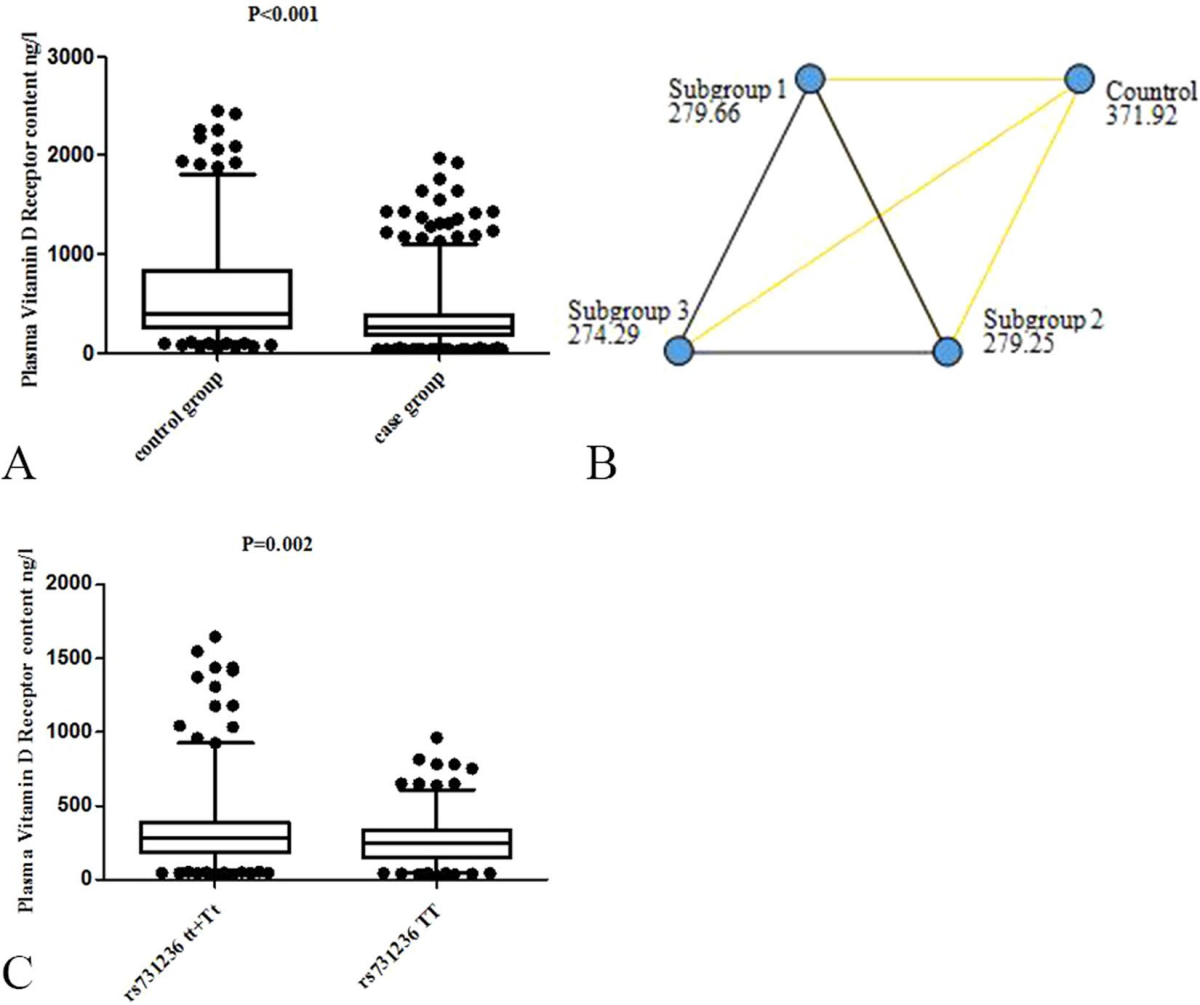
Plasma VDR levels in control (n = 485) and case (n = 454) groups, including subgroup 1 (n = 266), subgroup 2 (n = 105) and subgroup 3 (n = 83). (**A**) Plasma VDR levels was significant higher in the control group than those in the case group (P < 0.01). (**B**) Plasma VDR levels was significant higher in the control group than those in the subgroup 1, 2, and 3 respectively. Data represent the median, the unit is ng/L. The yellow line indicates a statistically significant difference (P < 0.05) and the black line indicates no statistically significant difference (P > 0.05). (**C**) Plasma VDR levels was significant higher in patients with rs731236 tt/Tt genotypes (n = 259) than those in the patients with TT genotype (n = 195). The line in the box represents the median. The interval of box is the 25th and 75th percentiles. The interval of whiskers is the 5th and 95th percentiles.

The plasma VDR levels of the case and control groups are shown in Fig. 1. The median plasma VDR content was 371.92 ng/L (range 50.98–2450.68 ng/L) in control group, 277.56 ng/L (range 27.61–2262.17 ng/L) in case group, 279.66 ng/L (range 27.61–2262.17 ng/L) in subgroup 1, 279.25 ng/L (range 35.68–1646.20 ng/L) in subgroup 2 and 274.29 ng/L (range 36.67–2225.92 ng/L) in subgroup 3. The plasma VDR levels in control group were significantly higher than that in case group (Fig. 1A) and all subgroups (Fig. 1B). Low plasma VDR levels were identified as associated with LDD (P < 0.001). There were no significant difference between all subgroups (all P > 0.05) (Fig. 1B). Regarding *TaqI* polymorphism, we found that plasma VDR levels in cases with TT genotype were significantly lower than those of tt + Tt (P = 0.002) (Fig. 1C).

### VDR expressions and LDD

**Figure 2:**
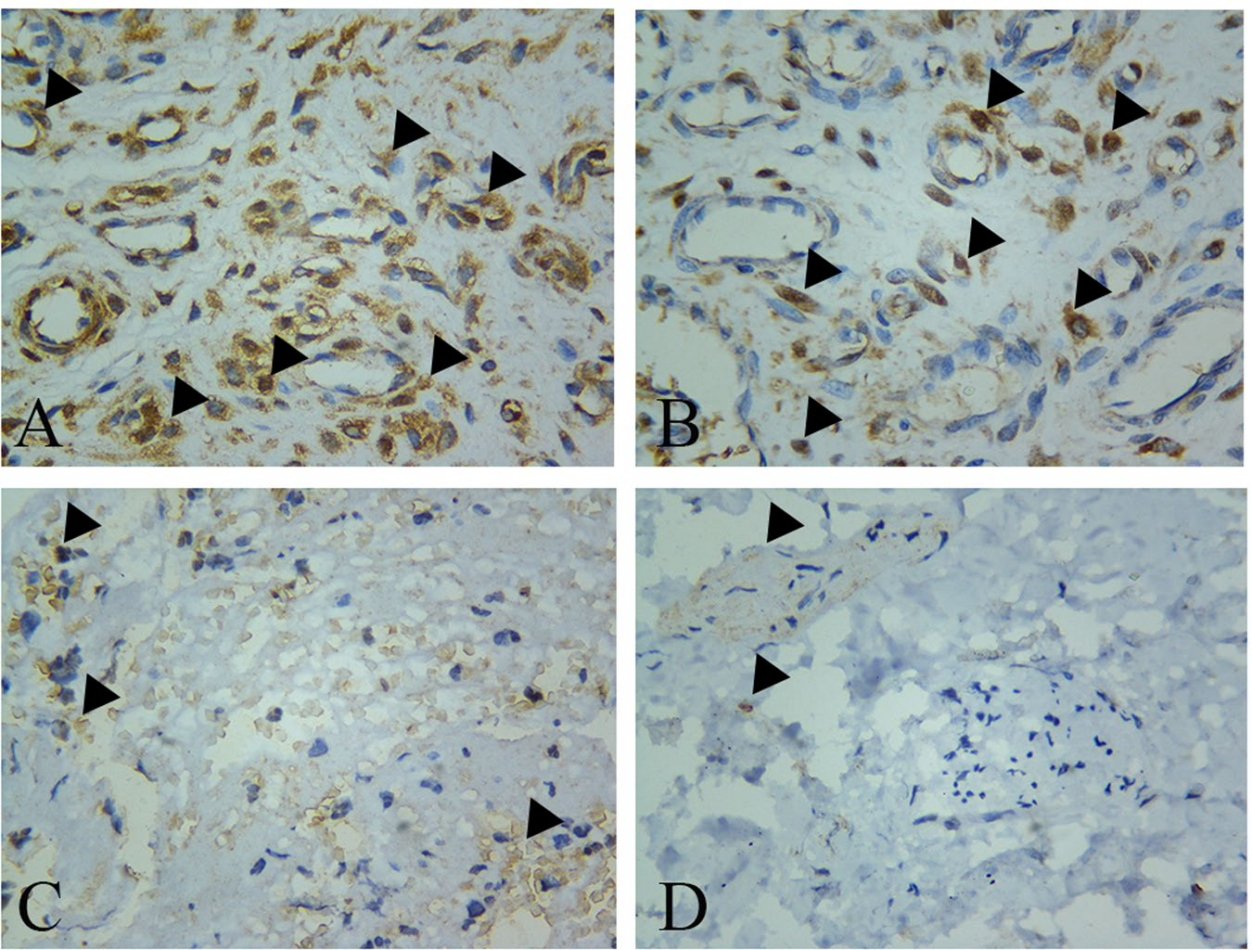
VDR expression was analyzed by immunohistochemistry assay. Expression of VDR was higher in the control group (**A**,**B**) than that in the case group (**C**,**D**). (Magnification, 400×) Black arrowheads show VDR positive cells.

As shown in Fig. 2, VDR expressed in the nucleus and cytoplasm as well as outside the cell was detected in the nucleus pulposus of the normal disc by IHC. VDR expression was notably decreased in degenerated disc compared with those in normal disc. Significant statistical difference was found in integral optical density (IOD) analysis between the case and the control groups (4,296.58 ± 2,212.70 vs. 228,492.47 ± 52,995.05, P = 0.001).

## Discussion

In the current study, the results showed that the *TaqI* (rs731236) polymorphism in VDR gene was associated with a predisposition to LDD in all genetic model comparisons. After dividing the LDD patients into three subgroups, we found a significant association between *TaqI* polymorphism and lumbar disc herniation (subgroup 1). Furthermore, low plasma VDR levels and decreased VDR expressions were found in LDD patients. To the best of our knowledge, this is the first study to investigate the associations between VDR polymorphisms, plasma VDR levels and VDR expressions and LDD.

The association between *TaqI* polymorphism and LDD was first investigated in Finnish population^32^. A Japanese study confirmed that Tt genotype of *TaqI* polymorphism was more frequently associated with LDD, but no tt genotype was found in their subjects^17^. Another study reported that the t allele of *TaqI* polymorphism was associated with a high risk of LDD^14^. However, several studies suggested no association between *TaqI* polymorphism and LDD^18,31,33–35^. Our study confirmed that the *TaqI* polymorphism was associated with susceptibility to LDD and indicated that the T allele may increase the risk of LDD. It is partly consistent with prior studies in Asian and Italian cohorts^15,23^. There are several possible explanations for the presence of heterogeneity. First, different diagnostic techniques may lead to differences. For example, diagnosis criterion was based on CT scans in Yuan’s study^18^, whereas diagnosis criterion was based on MRI in Noponen-Hietala’s study^34^. Second, different studies used variable radiographic features to define the degenerative disc disease phenotypes may be affect genetic association^36^. Third, the number of participant and the ethnicity of the study population might change the results of genetic association.

*TaqI* polymorphism was found in the 3′ UTR of VDR gene^27^. The 3′ UTR has been found to have the function of controlling gene expression by affecting the localization, stability and translation of mRNAs^29,30^. Changes in mRNA may affect the function and expression of protein. The relationship between *TaqI* polymorphism and VDR protein has not been reported. In the current study, we found that plasma VDR levels were lower in LDD patients than those in the controls. Correspondingly, the results of IHC analysis showed that decreased VDR expression level were found in the degenerated disc. These results indicated that lower VDR expression levels represented a risk factor for LDD. Moreover, we further evaluated whether the genetic polymorphism can influence plasma levels of VDR. Compared with rs731236 tt/Tt genotypes, rs731236 TT genotype was associated with lower levels of VDR expression. Taken together, these findings indicate that rs731236 TT genotype were a genetic risk factor for development of LDD, probably by decreasing the expression of VDR. In addition, we found the wide distribution of ELISA data in the current study. This may be due to the fact that our study is hospital-based case-control study. All participants were recruited from the same hospital. The selection bias could not be ruled out. Secondly, a wide range of ages in case and control groups may also lead to the wide distribution of ELISA results. VDR is widely expressed in various tissues and cells of the human body. Low plasma VDR levels were found in some degenerative musculoskeletal condition, such as osteoporosis^37,38^. Previous studies of animal models showed that the lack of VDR signaling may lead to proinflammatory monocyte phenotypes associated with increased inflammatory response, cartilage damage, and bone erosion^11^. It seems to indicate that VDR plays an essential role in development of LDD. The real influence of VDR on the molecular mechanism of LDD requires further research.

There are several limitations in our study. First, small size of the sample may weaken the statistical power. Further large scale studies are required to validate our findings. Second, potential selection bias could be introduced into a hospital-based case-control study, which may inevitably affect the result to some degree.

## Conclusions

The current study showed that VDR *TaqI* (rs731236) polymorphism was associated with a predisposition to LDD. Plasma VDR and VDR expression levels may be the marker for the occurrence and development of LDD.

## Materials and Methods

### Subjects

The study was approved by the Ethics Committee of the First Affiliated Hospital of Guangxi Medical University. All the experimental protocol and the methods were carried out in accordance with the relevant guidelines and regulations, and complied with the principles of the Declaration of Helsinki. Written informed consent was achieved from each participant.

**Figure 3:**
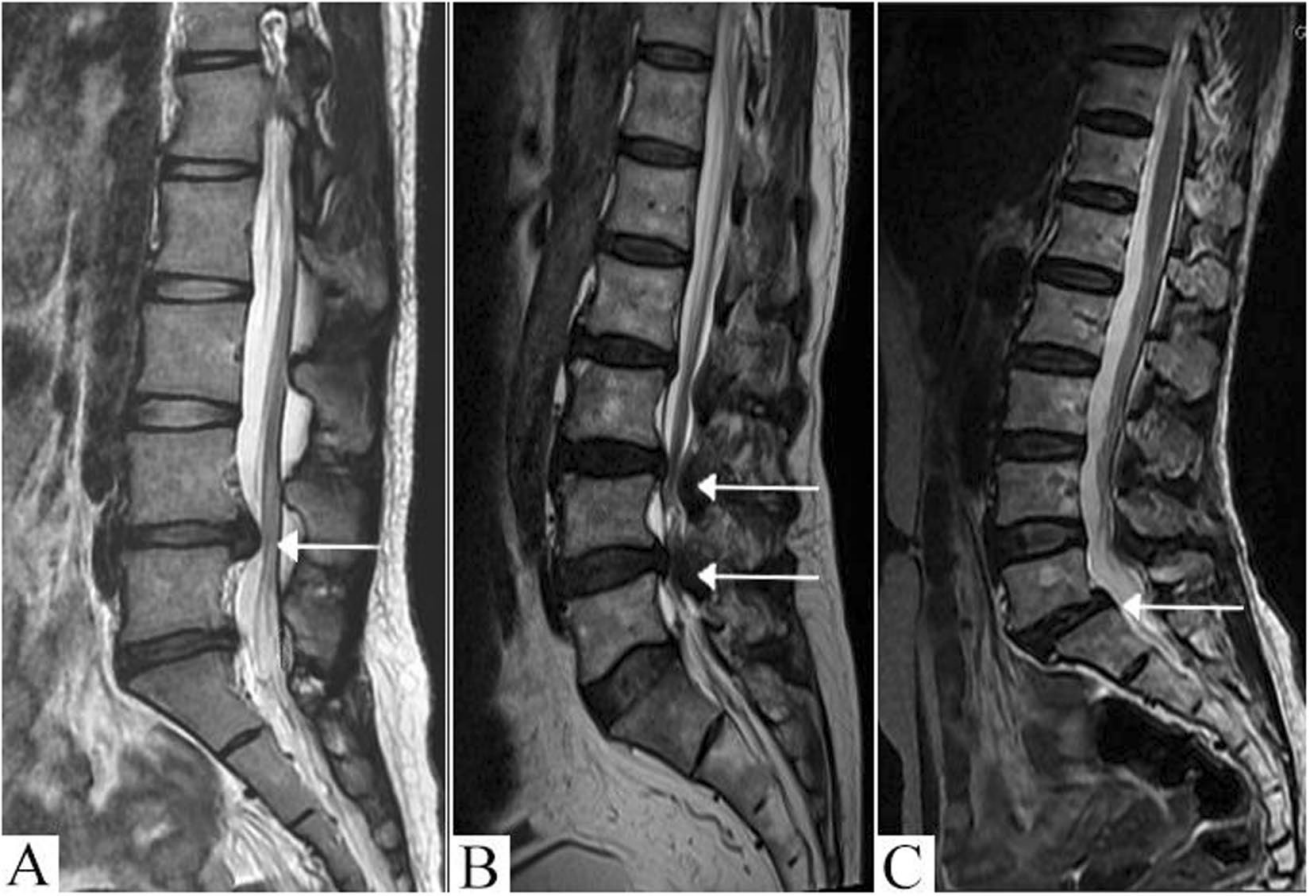
Classification of patients into subgroups by MRI imaging. (**A**) Subgroup 1: patients with lumbar disc herniation; white arrow indicates L4/5 herniated disk bulges out toward the spinal canal. (**B**) Subgroup 2: patients with lumbar spinal stenosis; white arrows indicate L3-4 level spinal stenosis caused by degenerative thickening of the ligamentum flavum. (**C**) Subgroup 3: patients with lumbar spondylolisthesis; white arrow indicates subluxation of L5 vertebral body relative to S1 vertebral body.

This study participant including 454 patients with LDD and 485 controls were recruited from Spine Surgery and Physical Examination Center, the First Affiliated Hospital of Guangxi Medical University. The control group was composed of 252 females and 233 males, and the case group was composed of 259 females and 195 males. All patients were diagnosed with LDD based on clinical examinations and Magnetic Resonance Imaging (MRI). Clinical examinations were performed by one attending spine surgeon. MRI images were obtained using a 1.5-T magnetic resonance imaging Achieva scanner (Philips Medical Systems; Best, the Netherlands) with Nova Dual gradients. The following inclusion criteria were applied: (1) low back pain as the main symptom for at least 3 months; (2) MRI shows degenerative changes in lumbar spine; (3) no previous spinal surgery or other treatment that would deform the lumbar spine. Evaluation of the characteristics of the phenotypes based on MRI was performed by two independent radiologists. Any dispute between the two radiologists was resolved by a senior radiologist. According to MRI phenotypes^36^, the patients with LDD were further divided into three different mutually exclusive subgroups based on as follows: subgroup 1 included 266 patients affected by lumbar disc herniation; subgroup 2 included 105 patients affected by lumbar spinal stenosis and subgroup 3 included 83 patients affected by lumbar spondylolisthesis (Fig. 3). The exclusion criteria were applied: (1) a history of clinician-diagnosed low back pain at least six months’ duration that was present more than half the days of the month; (2) spine deformity; (3) the history of intraspinal tumor, trauma, inflammatory disease and rheumatoid arthritis; (4) previous spinal surgery; (5) MRI phenotype of one patient fit into more than one subgroup. To be eligible for control group, the subjects had no history of low back pain, and were screened by a 1.5-T lumbar spine MRI scan. Disc degeneration were identified in the MRI images and graded according to the modified Pfirrmann grading system^39^. The subjects with Pfirrmann’s Grade 1 were included in control group. Furthermore, we collected degenerative disc tissues (n = 34) and normal disc tissues (n = 21) from patients with lumbar disc herniation (subgroup 1) and patients with traumatic lumbar vertebral fracture, respectively. Patients with traumatic lumbar fracture had no history of low back pain before surgery and MRI evaluation showed no significant disc degeneration. According to Schneiderman’s classification^40^, Grade 1 was in 19 patients and Grade 2 was in 2 patients. These samples were used to evaluate VDR expression via immunohistochemistry (IHC).

### Genotyping

Peripheral blood samples were taken from the study participant and stored at −80 °C for laboratory analysis. Genomic DNA from LDD patients and the controls were extracted from peripheral blood using Whole Blood DNA Isolation Kit (Promega, Madison, WI, USA). Three single nucleotide polymorphisms (SNPs) in VDR gene were selected including *FokI* (rs2228570), *ApaI* (rs7975232) and *TaqI* (rs731236). The SNPs genotyping were analyzed by real-time PCR using Taq PathTM ProAmpTM Master Mixes (A30867, Thermo Fisher Scientific, Carlsbad, CA, USA) and TaqMan® SNP Genotyping Assay (4351376, Thermo Fisher Scientific, Carlsbad, CA, USA) and performed in LightCycler®96 system (LIGHTCYCLER, Mannheim, Germany). Thermal cycling used the following conditions: pre-read at 60 °C for 30 seconds, initial denaturation at 95 °C for 5 minutes, denaturation at 95 °C for 15 seconds, annealing at 60 °C for 1 minute and finally post-read at 60 °C for 30 seconds. Two authors independently performed the analysis of the genotype results.

### Plasma VDR levels

Plasma VDR levels were detected by using Enzyme-linked Immunosorbent Assay (ELISA) kit (MyBioSource, San Diego, CA, USA). According to the kit specification, the sample was diluted with the sample dilution provided by the kit to 1/5 of the original concentration. All steps in this experiment were performed strictly in accordance with the manufacturer’s instructions. We measured the absorbance (OD) of each well at 450 nm by the microplate reader (ELx800; BioTek Instruments, Inc., Winooski, VT, USA). According to the corresponding value of the standard, we drew the standard curve. Finally, we calculated the concentration of samples by the standard curve.

### Immunohistochemistry (IHC)

Disc tissues after the surgery to remove were fixed with 4% formaldehyde and embedded in paraffin for serial sectioning at 5 μm. After dewaxing and dehydration, the sections were immersed in 0.01 mol/L citrate buffer (pH = 6) for autoclaving antigen repair. The sections were incubated in 3% hydrogen peroxide for 10 min and subsequently with 5% BSA blocking solution for 10 min at room temperature. Primary antibody (ab137371, Abcam, Cambridge, UK) was diluted at 1:300 and incubated overnight at 4 °C, followed by secondary antibody (ab150077, Abcam, Cambridge, UK) at 37 °C for 15 min. DAB reagent was added to the section that was examined by microscope and incubated at room temperature for 5 min. Sections were counterstained with hematoxylin for 1 min, dehydrated through several baths of graded hydrochloric-alcohol and xylenes and then coverslipped using mounting solution. Finally, slices were observed using the Aperio System (Leica), in which 5 typical fields were randomly selected for analysis (400× magnifications). The integral optical density (IOD) of VDR-positive expression in each image was calculated by the professional image analysis software, Image-Pro Plus 6.0 (Media Cybernetics, Rockville, MD, USA).

### Statistical analysis

All the data analysis were performed by using SPSS version 20.0 (SPSS Inc., Chicago, IL, USA). Kolmogorov-Smirnov test was used to assess the normality of data distribution. The Mann-Whitney test was utilized to examine the differences of age, BMI and the plasma VDR levels between case group including subgroups and control group. The independent-sample t-test was used to examine the VDR expression levels between case and control groups in IHC. The differences in allelic frequencies and genotype distributions were compared using the chi-square (χ^2^) test. The P value was adjusted to the corresponding original 5% level by dividing by the number of comparisons to the control group. Hardy-Weinberg equilibrium was measured using a goodness-of-fit χ^2^ test. The ORs and 95% CIs were calculated to estimate VDR gene polymorphisms and LDD risk. Logistic regression was used to evaluate effects of confounders such as age by obtaining adjusted ORs and 95% CIs for genotypes and alleles. Statistical significance was set at P < 0.05.
